# SEOM Clinical Guideline for treatment of muscle-invasive and metastatic urothelial bladder cancer (2016)

**DOI:** 10.1007/s12094-016-1584-z

**Published:** 2016-11-29

**Authors:** M. Lázaro, E. Gallardo, M. Doménech, Á. Pinto, A. González del Alba, J. Puente, O. Fernández, A. Font, N. Lainez, S. Vázquez

**Affiliations:** 1 Medical Oncology Department, Hospital Álvaro Cunqueiro-Complexo Hospitalario Universitario de Vigo, Estrada Clara Campoamor, 34136312 Vigo, Spain; 2 Medical Oncology Department, Parc Taulí Sabadell Hospital Universitari, Sabadell, Spain; 3 Medical Oncology Department, Althaia, Xarxa Assisencial i Universitària de Manresa, Manresa, Spain; 4 Medical Oncology Department, Hospital Universitario La Paz-Idipaz, Madrid, Spain; 5 Medical Oncology Department, Hospital Universitario Son Espases, Palma de Mallorca, Spain; 6 Medical Oncology Department, Hospital Universitario San Carlos, Madrid, Spain; 7 Complexo Hospitalario Universitario de Ourense, Badalona, Spain; 8 Medical Oncology Department, Germans Trias i Pujol University Hospital, Badalona, Spain; 9 Medical Oncology Department, Complejo Hospitalario de Navarra, Pamplona, Spain; 10 Medical Oncology Department, Hospital Universitario Lucus Augusti, Lugo, Spain

**Keywords:** Bladder cancer, Cystectomy, Chemotherapy, Clinical guidelines

## Abstract

The goal of this article is to provide recommendations for the diagnosis and treatment of muscle-invasive and metastatic bladder cancer. The diagnosis of muscle-invasive bladder cancer is made by pathologic evaluation after transurethral resection. Recently, a molecular classification has been proposed. Staging of muscle-invasive bladder cancer must be done by computed tomography scans of the chest, abdomen and pelvis and classified on the basis of UICC system. Radical cystectomy and lymph node dissection are the treatment of choice. In muscle-invasive bladder cancer, neoadjuvant chemotherapy should be recommended in patients with good performance status and no renal function impairment. Although there is insufficient evidence for use of adjuvant chemotherapy, its use must be considered when neoadjuvant therapy had not been administered in high-risk patients. Multimodality bladder-preserving treatment in localized disease is an alternative in selected and compliant patients for whom cystectomy is not considered for clinical or personal reasons. In metastatic disease, the first-line treatment for patients must be based on cisplatin-containing combination. Vinflunine is the only drug approved for use in second line in Europe. Recently, immunotherapy treatment has demonstrated activity in this setting.

## Introduction

According to GLOBOCAN, about 430,000 new bladder cancer cases and 165,000 bladder cancer deaths occurred worldwide in 2012, making it the ninth most common type of cancer for both gender [1].

Europe has one of the highest incidence rates of bladder cancer in the world. According to cancer registry data, the highest incidence rates in men were reported in Southern Europe, particularly in Spain (age-standardized rate (ASR) = 36.7 per 100,000) and Italy (ASR = 33.2 per 100,000). In Spain, around 12,200 new cases are diagnosed every year, with 47 cases per 100,000 men and almost eight cases per 100,000 women [2].

Overall, bladder cancer mortality has been decreasing all over the world except in countries undergoing rapid economic transition [3]. Mortality rates in European men were by far the highest recorded worldwide (e.g., Spain: ASR = 8.2 per 100,000).

Smoking is recognized as the most important risk factor for urothelial bladder cancer (BC) (ever-smokers are considered to have a 2.5 times higher risk of developing this tumor than nonsmokers) [3] and is estimated to account for 50% of tumors (former tobacco smoking RR 2.04, 95% CI 1.85–2.25, *p* < 0.001; current tobacco smoking RR 3.47, 95% CI 3.07–3.91, *p* < 0.001 when compared to never smokers) [4].

Following smoking, occupational exposure to carcinogens, namely aromatic amines, polycyclic aromatic, hydrocarbons, and chlorinated hydrocarbons is viewed as the second most important risk factor for urothelial BC. There are several medical conditions that may predispose individuals to bladder tumorigenesis: chronic urinary retention and upper tract dilation increase urothelial exposure to carcinogens and carcinogenesis associated with chronic inflammation or schistosomiasis [2].

## Methodology

The SEOM guidelines have been developed with the consensus of ten genitourinary cancer oncologists from SEOM (Spanish Society of Medical Oncology) and SOGUG (Spanish Oncology Genitourinary Group).

To assign a level of levels of evidence and grades of recommendation we have used Table 1 [5].

**Table 1:** Levels of evidence/grades of recommendation

*Levels of evidence*	
I	Evidence from at least one large randomized, controlled trial of good methodological quality (low potential for bias) or meta-analyses of well-conducted randomized trials without heterogeneity
II	Small randomized trials or large randomized trials with a suspicion of bias (lower methodological quality) or meta-analyses of such trials or of trials with demonstrated heterogeneity
III	Prospective cohort studies
IV	Retrospective cohort studies or case–control studies
V	Studies without control group, case reports, experts opinions
*Grades of recommendation*	
A	Strong evidence for efficacy with a substantial clinical benefit, strongly recommended
B	Strong or moderate evidence for efficacy but with a limited clinical benefit, generally recommended
C	Insufficient evidence for efficacy or benefit does not outweigh the risk or the disadvantages; optional
D	Moderate evidence against efficacy or for adverse outcome, generally not recommended
E	Strong evidence against efficacy or for adverse outcome, never recommended

Statements without grading were considered justified standard clinical practice by the SEOM/SOGUG faculty and experts.

## Molecular biology: molecular classification

Molecular genetic evidence supports the existence of two distinct pathogenetic pathways for bladder cancer development: low-grade papillary superficial tumors (characterized by activation of the receptor tyrosine kinase-Ras pathway, and activating mutations in the HRAS and fibroblast growth factor receptor 3 (FGFR3) genes) and high-grade invasive BC (characterized by alterations in the p53 and retinoblastoma (RB1) pathways). These genes interact with the Ras-mitogen-activated protein kinase (MAPK) signal transduction pathways [6].

The variability in outcomes of muscle-invasive bladder cancer (MIBC) can be explained by differences in the genetic changes involved in bladder cancer development and progression. The Cancer Genome Atlas Project completed one of the most comprehensive molecular analyses in bladder cancer, examining 131 cases of MIBC [7]. Tumors were histologically categorized and evaluated via whole genome sequencing, whole exome sequencing, DNA copy number, complete mRNA and microRNA expression, DNA methylation, and protein expression and phosphorylation. Many genes were consistently mutated, including TP53, PIK3CA, RB1, FGFR3, and TSC1. In addition, a few pathways were identified as consistently dysregulated. Mutations in the p53/RB tumor suppressor pathway were seen in 93% of tumors, and alterations in the PI3K/AKT/mTOR and RTK/RAS signaling were seen in 72%. Finally, alterations that impact epigenetic changes were seen up to 89% of tumors.

In the last few years, several studies have proposed a molecular classification of bladder cancer based in the whole genome mRNA expression profiling. The molecular subtypes identified in bladder cancer have significant similarities with the molecular classification previously established in breast cancer patients [8]. A group from the University of North Carolina classified high-grade bladder cancer in luminal and basal-like tumors using a 47-gene signature (BASE 47) [9]. TCGA study [7] defined four mRNA expression-based subtypes (cluster I–IV). The cluster I–II correlated with luminal subtype and cluster III–IV with basal subtype. A third classification proposed by the MD Anderson group identified basal tumors and segregated the luminal subtype in luminal and p53 like tumors [10]. In an expanded cohort of 238 TCGA invasive bladder cancers, four subtypes were identified. Besides luminal and basal subtypes, two new subtypes were proposed related to immune cell infiltration termed “immune undifferentiated” and “luminal immune” characterized by the expression of immune genes (CTLA4 and CD274) [11].

Intrinsic subtypes in MIBC patients are associated with specific clinical-pathological characteristics. Basal MIBC are more prevalent in women and are enriched with squamous pathological features, whereas tumors with a micropapillary histological variant are mainly classified in the luminal subtype [12] Basal tumors were associated with advanced disease with poor prognosis. In contrast, luminal tumors have papillary features that correspond to better outcome when presented at early stage.

Furthermore, molecular classification according to gene expression profile can be correlated with bladder cancer outcome and chemosensitivity. Luminal subtype in BC patients has been associated with chemoresistance. Moreover, only a minority of patients with p53 like tumors responded to neoadjuvant MVAC chemotherapy, suggesting that patients with this molecular subtype should be treated with alternative approaches [10]. In contrast, the greatest benefit of neoadjuvant chemotherapy has been observed in patients with basal subtype [13]. In addition, the evidence of immune cell infiltration in specific bladder cancer subtypes suggests a potential benefit of immune checkpoint agents in these patients. A recently published study showed higher efficacy of atezolizumab in advanced bladder cancer patients with the TCGA luminal cluster II subtype compared with other subtypes [14]. In conclusion, molecular classification of bladder cancer can be a useful tool to select the most adequate therapy for each patient. These advances should be incorporated into the design of clinical trials and progressively into the clinical practice.

## Diagnosis and staging

The symptom that is most frequently presented is hematuria, and irritative urinary symptoms are also common.

Initial diagnosis study is composed of physical examination, complete tests with hemograma and biochemistry, cytoscopy, urinary cytology and an image of the upper urinary tract. The findings of the cytoscopy must be described in detail, including location, size and number of injuries. The fluorescent cytoscopy can detect more tumors, especially in carcinoma in situ and papillary injuries, therefore it is useful in patients on whom a radiotherapy treatment is chosen for.

It is followed by bimanual examination under anesthesia (EUA) and transurethral resection of the bladder (TURBT). This is important to obtain a pathological diagnosis and for MIBC in staging, therefore, it is mandatory to include a representation of the muscular layer. In cases with positive cytology and a normal cystoscopy, an upper urinary tract and prosthetic urethra exam must be carried out.

Histological diagnosis is based on the World Health Organization (WHO) classification [15] and tumor grade is an important factor to determine the potential progression and recurrence of the tumor.

In patients with a MIBC confirmed diagnosis, a computed tomography (CT) of chest, abdomen and pelvis, must be performed as a method of initial staging, including an excretory-phase CT urography for the upper urinary tract study, being MRI also an option. Both are useful for local staging. Routine FDG-PET/CT is not recommended for routine initial staging on MIBC. Bone scan must be performed if there are bone related symptoms or high levels of alkaline phosphatase [16].

**Table 2:** TNM staging

TNM staging system [62]
Stage I: T1 N0 M0
Stage II: T2a–T2b N0 M0
Stage III: T3a–T3b, T4a N0 M0
Stage IV: T4b N0 M0
Any T: N1-N3 M0
Any T: Any N M1

Staging must be done according to the norms of the American joint committee on cancer (AJCC) staging manual 7th edition (Table 2).

### Recommendations

Initial diagnosis study is composed of physical examination cytoscopy, urinary cytology and an image of the upper urinary tract, followed by bimanual EUA and TURBT.

Level of evidence 2. Grade of recommendation B.

CT of chest, abdomen and pelvis, must be performed as a method of initial staging, including an excretory-phase CT urography for the upper urinary tract study.

Level of evidence: 2. Grade of recommendation B.

## Prognostic factors

The relevance of prognosis classifications is to identify subgroups of patients to decide the most adequate management for every single patient and should be considered for stratification for future trials.

Tumor histologic grade and staging have been recognized as significant prognostic factors for recurrence and progression among patients with nonmetastatic bladder cancer. In addition, pathological response has been proved to be prognostic for patients with muscle-invasive bladder tumors treated with neoadjuvant chemotherapy [17].

At metastatic setting, a prognostic classification was established for first-line chemotherapy after identifying two independent prognostic clinical factors: Karnofsky performance status less than 80% and the presence of visceral metastases (liver, lung and bone) [18]. Patients can be classified as good, intermediate or bad prognosis after the presence of none, one or both factors (median overall survival: 33, 13.4 and 9.3 months, respectively). This classification was subsequently validated in another independent series from a prospective study [19].

For second-line patients, another prognostic classification was proposed [20]. Three independent prognostic factors in this setting were identified: ECOG performance status more than 0, hemoglobin value less than 10 g/dL and the presence of liver metastases. After internal and external validation, four categories were established from the presence of none, one, two or three factors with median survival times of 14.2, 7.3, 3.8, and 1.7 months. Time from first-line chemotherapy [21] and albumin levels [22] have also proved independent prognostic significance and external validation.

At present, no prognostic biomarkers have been validated for these patients. The indefectible progression after standard chemotherapy and the introduction of new therapeutic approaches deserves further research efforts in this way.

### Recommendations

Use of prognostic classification in first-line chemotherapy.

Level of evidence: II. Grade of recommendation: B.

Use of prognostic classification in second-line chemotherapy.

Level of evidence: II. Grade of recommendation: B.

## Stage II–III treatment

### Radical cystectomy

Radical cystectomy (RC) with extended lymphadenectomy, often preceded by neoadjuvant cisplatin-based chemotherapy, and urinary diversion is the gold standard definitive surgical treatment MIBC [23] and involves removal of the bladder, prostate, seminal vesicles, proximal vas deferens and proximal urethra in men, and bladder, uterus, ovaries, fallopian tubes, urethra and part of vagina in women. A randomized [24] trial of robotic-assisted laparoscopic versus open radical cystectomy showed no difference in morbidity or length of hospital stay, but longer operative time and increased cost in the robotic group, similar to results from the CORAL study [25] from the UK. Forms of urinary diversion include orthotopic bladder replacement and uretero-ileo-urethrostomy (Studer and Padovana neobladder) or an incontinent external ostomy with cutaneous ureteroileostomy (Bricker diversion). A glomerular filtration rate of at least 50 mL/min is mandatory for continent reservoirs since the kidneys must compensate the metabolic acidosis following incorporation of bowel in the urinary tract [26]. Candidates for a continent urinary diversion should also have normal liver function (risk of hyperammonemia if the reservoir becomes infected), and should not have undergone any previous major bowel resection in the ileocecal area (risk of vitamin B12 deficiency) [26].

Three retrospective cohort studies found regional lymph node dissection to be associated with a lower risk of mortality compared with no lymph node dissection in individuals undergoing radical cystectomy for localized muscle-invasive bladder cancer [27–29]. More extensive lymph node dissection was associated with a decreased risk of all-cause or bladder cancer-specific mortality [27]. In one study, dissection in which ten lymph nodes were removed was found to be associated with lower 10 year overall (30.3 vs 39.4%; *p* < 0.001) and cancer-specific (38 vs 46%) mortality compared with dissection in which <10 lymph nodes were removed [27].

### Recommendations

Radical cystectomy with extended lymphadenectomy is the gold standard definitive surgical treatment in muscle-invasive bladder cancer.

Level of evidence: III. Grade of recommendation: A.

### Neoadjuvant and adjuvant treatment

Although surgery may be curative, a large proportion of patients will develop recurrence and will ultimately die of metastatic disease. Several studies have been performed with perioperative chemotherapy, in both neoadjuvant and adjuvant settings.

The major impediment to the use of perioperative chemotherapy in patients with bladder cancer is renal impairment and comorbidities. Around 50% of patients have a glomerular filtration rate of less than 60 mL/min, making them ineligible for cisplatin treatment.

#### Neoadjuvant treatment

Neoadjuvant chemotherapy has been evaluated in patients with clinical stage T2-T4aN0M0 MIBC who are candidates for RC or definitive radiotherapy. The rationale for giving chemotherapy before cystectomy or full-dose radiation therapy is to treat micrometastases present at diagnosis, as approximately 50% of patients diagnosed with MIBC develop metastatic disease within 2 years.

Cisplatin-based combination chemotherapy provides a greater survival benefit than surgery alone in two large, well-designed, randomized trials [17, 30] and two meta-analyses [31, 32]. Despite a 5% survival benefit at 5 years, the neoadjuvant approach prior to cystectomy has not been widely accepted.

The different neoadjuvant regimens have not been compared in randomized trials, and the ideal cisplatin-based combination chemotherapy has not been established. Methotrexate, vinblastine, doxorubicin, and cisplatin (MVAC) is the best-studied regimen for neoadjuvant chemotherapy. Dose-dense MVAC (DDMVAC) and gemcitabine plus cisplatin (GC) regimens have been also evaluated in several retrospective studies with no substantive difference in the response rate.

### Recommendation

Neoadjuvant chemotherapy is recommended for T2–T4a, cN0 M0 bladder cancer and should always be cisplatinum-based combination therapy.

Level of evidence I. Grade of recommendation A.

Neoadjuvant chemotherapy is not recommended for patients with ECOG PS 2 and/or impaired renal function.

Level of evidence I. Grade of recommendation A.

#### Adjuvant treatment

An updated meta-analysis of nine randomized trials, including 945 patients found and overall survival (OS) and disease-free survival (DFS) benefit among those who received cisplatin-based adjuvant chemotherapy [33]. For OS the pooled hazard ratio (HR) across all nine trials was 0.77 (95% CI 0.59–0.99; *p* = 0.049). On the other hand, the pooled HR for DFS was 0.66 (95% CI 0.45–0.91; *p* = 0.014). This DFS benefit was more apparent among those with positive nodal involvement (*p* = 0.010).

All the published trials were prematurely terminated and all included enrolled less than 100 patients each. Moreover, two larger randomized clinical trials reported conflicting results (Spanish trial SOGUG 99/01 that enrolled 142 patients [34] and Italian trial that accrued over 180 patients [35]). In the SOGUG trial, HR for intention-to-treat population was 0.37 (95% CI 0.64–0.22, *p* < 0.0004) favorable to chemotherapy arm, but the study was prematurely closed. In the Italian trial was no differences in overall survival between adjuvant chemotherapy and control group (48% at 5 years).

In the largest and most recent trial, 284 patients were randomly assigned to either four cycles of adjuvant chemotherapy (immediate treatment) or deferred treatment. The difference in 5-year overall survival was not statistically significant (53.6 vs 47.7%; HR 0.78, 95% CI 0.56–1.08) [36]. In a post hoc exploratory analysis, overall survival was significantly improved in those without lymph node involvement at baseline (79.5 vs 59.0%).

### Recommendation

While there is still insufficient evidence for the routine use of adjuvant chemotherapy in clinical practice, it is likely that high-risk patients (extravesical and/or node-positive disease) that have not received neoadjuvant chemotherapy will benefit most from adjuvant chemotherapy.

Level of evidence I. Grade of recommendation A.

Neoadjuvant chemotherapy should still be preferred due to a higher level of evidence and better feasibility.

Level of evidence I. Grade of recommendation A.

### Bladder-sparing treatments

Bladder-preserving approaches are reasonable alternatives to cystectomy for patients who are unfit for surgery and those who wish to avoid radical surgery.

Clinical criteria helpful in selecting patients for bladder preservation include small tumors size (≤5 cm) without carcinoma in situ, visible complete TURBT, early tumor stage (T2–T3a), no hydronephrosis and no metastatic lymphadenopathy [37].

Options include TURBT alone, TURBT followed by radiotherapy alone, chemotherapy alone or combination of chemotherapy and radiotherapy (trimodality treatment). However, only chemotherapy combined with radiotherapy has been evaluated in prospective randomized comparisons, the other treatment options are still considered to be investigational.

#### Trimodality treatment

Trimodality treatment (TMT) include maximal TURBT followed by radiation (40–45 Gy to the pelvis) with concurrent radiosensitizing chemotherapy and additional radiation boost to the bladder (20–25 Gy) if complete response is documented on repeat biopsy. If persistent or recurrent disease is observed at response evaluation o during follow-up, salvage cystectomy is recommended.

Three prospective randomized trials have demonstrated that concurrent chemoradiotherapy is superior to radiotherapy alone. In a randomized trial of 360 patients, radiotherapy with concurrent mitomycin C and 5-fluorouracil improved 2 year locoregional disease-free survival from 54% (radiotherapy alone) to 67%, and 5-year overall survival from 35 to 48% without increasing grade 3–4 acute or late toxicity [38]. In other study, 99 patients were randomized to receive radiation with or without cisplatin and demonstrated an improved local control rate when cisplatin was given [39]. In the third study, 333 patients were randomized to receive radiotherapy alone or radiotherapy plus carbogen and nicotinamide (CON). At 3 years, there was a 13% improvement in overall survival in favor of combination arm with a 14% lower risk of death [40]. The optimal combination of radiotherapy and chemotherapy has not been established.

Neoadjuvant chemotherapy in TMT has not been shown to improve survival. A phase III trial compared the efficacy of two cycles of cisplatin, methotrexate and vinblastine followed by concurrent chemoradiotherapy vs concurrent chemoradiotherapy alone. No difference in complete clinical response or 5-year overall survival was observed [41].

Results from several prospective trials have demonstrated the effectiveness of this approach. Five prospective RTOG trials of TMT in 468 patients demonstrates long term outcomes with 5 year and 10-year survival rates of 57 and 37%, respectively, 80% of patients retained an intact bladder at 5 year with low rates of toxicity [42].

### Recommendations

TURBT alone or radiotherapy alone cannot be recommended as standard treatment.

 Level of evidence: II. Grade of recommendation: B.

Trimodality treatment is an alternative in well- informed and compliant patients for whom cystectomy is not considered for clinical or personal reasons.

Level of evidence: I. Grade of recommendation: A.

## First-line therapy of locally advanced and metastatic disease

### First-line therapy for “fit” patients

Combination cisplatin-containing chemotherapy is the standard of care for fit first-line patients with advanced urothelial carcinoma. Both CG (cisplatin and gemcitabine) and MVAC (methotrexate, vinblastine, doxorubicin and cisplatin) are considered as acceptable treatment options in this indication. There are no clear differences in efficacy between these two schedules, but toxicity seems to be higher with MVAC. In a comparative randomized trial [43], median survival and 5-year survival were 14 months and 13% for GC, compared to 15.2 months and 15.3% for MVAC. However, CG has a better safety profile, with a lower incidence of febrile neutropenia, oral mucositis and alopecia, and therefore it is usually the preferred choice for first-line therapy.

High-dose intensity MVAC (HD-MVAC), administered every two weeks along with GCSF support, allows the delivery of twice the dose of cisplatin and doxorubicin. Compared with standard-dose MVAC [44], HD-MVAC showed a higher complete response rate (21 vs 9%; *p* = 0.009) and longer PFS (9.1 vs 8.2 months; *p* = 0.04), with fewer dose delays and a better toxicity profile, but without differences in OS. HD-MVAC should only be considered in selected populations and in centers with experience using this schedule, as it has not been compared with standard CG.

Adding a taxane to standard CG has also been explored in this setting. The combination of paclitaxel plus CG (PCG) did not show differences in PFS or OS in a phase III trial, and toxicity was higher for the triplet [45].

### First-line therapy for “unfit” patients

A significant percentage of patients with advanced urothelial cancer are considered “unfit” for cisplatin-based chemotherapy, based on the following criteria: Eastern Cooperative Oncology Group (ECOG) performance status (PS) 2 or Karnofsky index 60–70%; creatinine clearance <60 mL/min; loss of hearing and/or peripheral neuropathy ≥grade 2 according to the Common Terminology Criteria for Adverse Events version 4.0; or New York Heart Associations Class III heart failure [46]. There is no clear standard therapy for these patients, and the most usual procedure is changing cisplatin for carboplatin, therefore avoiding renal toxicity and improving tolerance to the schedule.

A phase II/III trial comparing the activity of carboplatin/gemcitabine (CaG) with that of carboplatin/methotrexate/vinblastine (CaMVi) in 178 unfit patients found similar efficacy for the two regimens [47], with a median OS of 9.3 and 8.1 months, respectively, and with a lower toxicity for CaG. Some other studies have tested the role of non-platinum combinations, such as paclitaxel–gemcitabine, which show encouraging activity, with response rates ranging 40–60%, but also considerable toxicity. Finally, for frail patients, monotherapy can also be an option, mainly with paclitaxel, gemcitabine or vinflunine, but with limited results.

### Recommendations

For first-line fit patients, both CG and MVAC are considered standard options. CG is preferred over MVAC mainly due to a better safety profile.

Level of evidence: 1. Grade of recommendation: A.

For first-line unfit patients, CaG should be considered the preferred treatment option.

Level of evidence: 1. Grade of recommendation: A.

### Second line

We have limited treatment options for this scenario. Most of the chemotherapy agents have been tested in phase 2 studies. Paclitaxel, docetaxel, oxaliplatin, pemetrexed, nab-paclitaxel, ifosfamida, among others, have a response rate of around 20%, without having proven a global survival benefit [48]. Combined use of chemotherapy agents increases the response rate and DFS [49].

Vinflunine, a third generation vinca alkaloid, showed a benefit in overall survival in eligible population during a phase 3 study, compared against best supportive care (BSC) (although not in the intended treatment population) and it has been approved by the EMA in this indication (IB) [50].

It is recommended, if possible, the inclusion of these patients in the clinical studies.

### Recommendations

For patients who progress after platinum based therapy offer vinflunine. Treatment in a clinical trial as an alternative.

Level of evidence I. Grade of recommendation B.

### New drugs and immunotherapy agents

Advances in the understanding of the molecular mechanisms of UBC have led to many studies to evaluate targeted therapies. Potential actionable genomic alterations, including activating mutations and RNA expression changes involving the PI3K/AKT/mTOR and RTK/RAS pathways have been frequently detected in the TCGA and other studies [7, 11, 51] Several studies have identified significant activity of targeted therapies in patients with these genomic alterations. For example, patients with mutations in PIK3CA (about 17% of tumors) can be sensitive to PI3K inhibitors, whereas patients with mutation/amplification of ERBB2 (about 9% of tumors) or ERBB3 mutations (about 6% of tumors) can respond to ERBB tyrosine kinase inhibitors, as has been recently demonstrated [52]. Moreover, inactivating mutations of genes involved in DNA repair pathways have also been identified in MIBC patients. Somatic mutations of ERCC2, a crucial gene of the nucleotide excision repair pathway, were found in 6–18% of urothelial tumors. These mutations have been associated with response to neoadjuvant cisplatin-based chemotherapy in MIBC patients [7, 11, 53, 54].

Immune checkpoint inhibition for cancer treatment is an area of growing research and recent studies have demonstrated that upregulation of PD-L1 is an important mechanism of immune escape in NMIBC. Overexpression of PD-L1 in UC correlates with high-grade disease and worse clinical outcome. Remarkable efficacy and safety was seen in a phase I expansion cohort of 67 patients with heavily pretreated metastatic bladder cancer. Patients received 15 mg/kg of MPDL3280A (atezolizumab), a human monoclonal antibody to PD-L1, every 3 weeks. 89 response rates were reported by PD-L1 positivity status, defined as 5% or higher of tumor-infiltrating immune cells staining for PD-L1 by IHC [55]. In this study, 27% of tumors were IHC 2- or 3-positive, as defined by expression of PD-L1 on tumor-infiltrating immune cells. The overall response rate for all patients by response evaluation criteria in solid tumors (RECIST) v1.1 was 26%, and was even more remarkable (43%) among patients with PD-L1+ tumor-infiltrating cells. Even among patients whose tumor-infiltrating immune cells were PD-L1−, the response rate was 11% as measured by RECIST v1.1. The median time to first response was 42 days (range 38–85 days). Based on these results, MPDL3280A received breakthrough designation by the FDA in June 2014. Recently, several other immunotherapies agents such nivolumab [56], pembrolizumab [57], avelumab [58] or durvalumab [59] have shown promising data in different phase I/II trials; and a phase II trial with atezolizumab in platinum-treated patients showed an ORR 16% (28% IC2/3 PDL1 subgroup) and an overall survival of 7.9 months (11.9 IC2/3 PDL1 subgroup) [60]. Another multiple PD-1/PDL-1 agents are currently being tested alone or in combination in advanced/refractory UC. Many more trials are in development in NMIBC.

## Follow-up

There is no data about the best follow-up strategy. Because most recurrences will develop within 24 months, the approach should be an oncological surveillance more intensive in this period of time: history, physical examination, urine cytology, liver and renal function tests, and electrolytes every 3 months for the first year, every 6 months for the second and third years, and then annually. CT imaging is reasonably performed every 6 months for the first 3 years (every 3 months if N+ the first year), then annually to year 5. After year 5 they should be performed only as clinically indicated [37, 61].

### Recommendation

For patients with MIBC a follow-up must be offered.

Level of evidence V. Grade of recommendation B.
